# “I didn’t have to prove to anybody that I was a good candidate”: a case study framing international bariatric tourism by Canadians as circumvention tourism

**DOI:** 10.1186/s12913-018-3385-2

**Published:** 2018-07-20

**Authors:** Carly Jackson, Jeremy Snyder, Valorie A. Crooks, M. Ruth Lavergne

**Affiliations:** Simon Fraser University, Blusson Hall 10516, 8888 University Drive, Burnaby, BC V5A 1S6 Canada

**Keywords:** Medical tourism, Canada, Bariatric surgery, Barriers

## Abstract

**Background:**

Medical tourism is a practice where patients travel internationally to purchase medical services. Medical tourists travel abroad for reasons including costly care, long wait times for care, and limited availability of desired procedures stemming from legal and/or regulatory restrictions. This paper examines bariatric (weight loss) surgery obtained abroad by Canadians through the lens of ‘circumvention tourism’ – typically applied to cases of circumvention of legal barriers but here applied to regulatory circumvention. Despite bariatric surgery being available domestically through public funding, many Canadians travel abroad to obtain these surgeries in order to circumvent barriers restricting access to this care. Little, however, is known about why these barriers push some patients to obtain these surgeries abroad and the effects of this circumvention.

**Methods:**

Semi-structured phone interviews were conducted with 20 former Canadian bariatric tourists between February and May of 2016. Interview questions probed patients’ motivations for seeking care abroad, as well as experiences with attempting to obtain care domestically and internationally. Interviews were digitally recorded, transcribed verbatim, and then thematically analyzed.

**Results:**

Three key barriers to access were identified: (1) structural barriers resulting in limited locally available options; (2) strict body mass index cut-off points to qualify for publicly-funded surgery; and (3) the extended wait-time and level of commitment required of the mandatory pre-operative program in Canada. It was not uncommon for participants to experience a combination, if not all, of these barriers.

**Conclusions:**

Collectively, these barriers restricting domestic access to bariatric care in Canada may leave Canadian patients with a sense that their health care system is not adequately addressing their specific health care needs. In circumventing these barriers, patients may feel empowered in their health care opportunities; however, significant concerns are raised when patients bypass protections built into the health system. Given the practical limitations of a publicly funded health care system, these barriers to care are likely to persist. Health professionals and policy makers in Canada should consider these barriers in the future when examining the implications medical tourism for bariatric surgery holds for Canadians.

## Background

Medical tourism is the act of crossing international borders with the intention of seeking medical care that is paid for out-of-pocket and beyond the scope of government administered cross-border care arrangements [1, 2]. There are many reasons why an individual may opt to seek medical care abroad. Some reasons include, but are not limited to, long wait times for care at home, more affordable price points for procedures abroad compared to domestic prices, restricted access to care domestically for legal or regulatory reasons, as well as structural barriers limiting availability of desired procedures domestically [2–6].

The umbrella term medical tourism may encompass many forms and scholars have generally delineated the types of medical tourism on the basis of legality of the patients’ desired procedures, as well as domestic availability. In particular, a form of medical tourism exists which encompasses patients travelling for medical services that are illegal, unavailable, or limitedly available due to regulatory restrictions in the patient’s home country but legal and readily available abroad. This form of medical tourism has been termed ‘circumvention tourism’ as it is characterized by patients circumventing restrictions in the health care system in their home country by either side-stepping domestic laws or bypassing administrative barriers that result in care being unavailable or limited domestically [7]. This particular form of medical tourism will be the focus of this subsequent analysis. Specifically, we will be presenting a case study examining how Canadians seeking bariatric (weight loss) surgery abroad, commonly known as bariatric tourism, represents a form of circumvention tourism in which these patients are bypassing barriers within the Canadian health care system that appear to be preventing access to this type of care domestically. First, this background will outline defining features and concerns associated with circumvention tourism and then second, will present an overview of the current bariatric landscape in Canada.

Most of the current conceptualizations of circumvention tourism have been explored in the literature through the legal classification of the procedure, focusing on travel abroad for care that is illegal in the patient’s home country. Some of these examples include assisted suicide, abortion, fertility tourism, female genital mutilation, and experimental treatments such as stem cell therapy [5, 7–10]. Much of this literature focuses on the legal ramifications on patients travelling to engage in these practices. For example, questions are asked such as: can (and should) home countries criminalize citizens who travel abroad to engage in care that is illegal at home but legal elsewhere [7, 9].

Despite the tendency to focus on treatments that are barred legally in the medical tourist’s home country, medical tourist activities that mirror those seen in the traditional circumvention tourism literature may arise from individuals attempting to access care in countries where procedures are legal but where regulatory and/or administrative barriers restrict access. For example, within Canada’s publicly funded health care system, rationing of care and/or resources takes place and therefore access can be limited or strict eligibility criteria may need to be met in order to access care [11]. Potential regulatory and/or administrative barriers work to restrict access to care on behalf of the health system as a means to ration resources to those most in need or those with the highest potential to be successful with the procedure [12]. Lack of access to care domestically due to these regulatory restrictions or administrative barriers, either perceived or real, can prompt individuals to circumvent the domestic health care system. While these barriers may not be legal in nature, it does appear that quite often, the barriers that prompt individuals to circumvent their domestic health care system are designed to protect patient health or to ensure the most efficient use of limited health care resources. When these restrictions and/or barriers are critically examined, it appears that circumvention behaviours occur in a relevantly similar fashion to previous understandings of circumvention tourism.

Through a qualitative examination of Canadians seeking bariatric services abroad and thus outside of the Canadian health care system, we seek to apply the concept of circumvention tourism for procedures that are legal but inaccessible due to regulatory or structural barriers. As such, this analysis will examine the distinctive challenges this practice can pose. When examining this broader conceptualization of circumvention tourism encompassing both legal and illegal procedures, additional procedure-specific ethical challenges can arise that have not previously been considered, especially given the legalistic focus of previous analyses. In order to explore how bariatric tourism by Canadians may be a form of circumvention tourism, the bariatric landscape in Canada must first be examined.

### A case of regulatory/administrative circumvention tourism

In Canada, there are currently approximately 5.3 million obese adults [13] and many of these individuals struggle with adequate weight control via conventional means of behaviour modification through diet and exercise. Consequently, bariatric surgery presents new opportunities for many of these individuals to treat their obesity. To qualify for bariatric surgery in Canada one must meet the minimum requirements set out by the National Institute of Health consensus conference in 1991, which requires an individual to have a body mass index (BMI) of 40 kg/m^2^ or a BMI of 35–39.5 kg/m^2^ with the presence of one or more significant co-morbidities such as type II diabetes, hypertension and/or obstructive sleep apnea [14, 15]. Additionally, Canada requires an individual to have a history of conventional methods of weight loss being ineffective at eliciting sustained weight loss. If a person does not meet these requirements, s/he will not be approved for surgery [16].

If a Canadian does meet eligibility requirements for bariatric surgery, they are eligible to enter the queue for publicly funded surgery. As each province or territory has their own provincial health care coverage, funding for specific procedures varies between them. In general, Canada only publicly funds three types of bariatric surgery: adjustable gastric banding, sleeve gastrectomy, and gastric bypass [16]. See Table 1 for a complete list of which provinces cover which procedures.

**Table 1:** Bariatric procedures covered by Canadian provinces and territories^a,b^

Province/Territory	Adjustable Gastric Banding	Sleeve Gastrectomy	Gastric Bypass
British Columbia	–	✔	✔
Alberta	✔	✔	✔
Saskatchewan	–	✔	✔
Manitoba	–	✔	✔
Ontario	–	✔	✔
Quebec	✔	✔	✔
Newfoundland and Labrador	✔	✔	✔
Nova Scotia	–	✔	–
Nunavut	–	–	–
Prince Edward Island*	–	✔	✔
New Brunswick*	✔	✔	✔
Northwest Territories	case-by-case	case-by-case	case-by-case
Yukon	case-by-case	case-by-case	case-by-case

*Procedures covered by provincial health care but contracted out of province

^a^Recreated from: [18, 16] [https://www.cihi.ca/en/bariatric-surgery-in-canada-infographic]

^b^Note: This table reflects coverage under Canadian public health care as of 2015. This table might not reflect what was available/publicly funded locally for participants in the study at their time of seeking surgery

The availability of bariatric surgery in Canada has been rapidly increasing. Canada has seen a three-fold increase in surgeries performed between 2006 and 2012: specifically, 1578 surgeries were performed in 2006 compared to 5989 performed in 2012 [16]. Despite the dramatic increase in surgery availability only about 1% of those potentially eligible based on BMI actually received the surgery through the public system^1^ [17]. This is, in part, attributable to the extensive pre-operative evaluation process that is associated with bariatric surgery in Canada, meaning that some of those that are eligible could be at various points in this multiyear process at any given time. More significantly, most eligible people will never opt to pursue this form of treatment. Structural barriers to access care contribute to very long wait times for bariatric surgery, with a national average wait time of just over 5 years, when studied in 2007 [15, 18].

The pre-operative evaluation process can contribute to delays in accessing bariatric surgery. In Canada primary care physicians act as gatekeepers to more specialized care [19] and a patient must receive a referral from their primary care physician in order to begin this evaluation process. Under the evaluation process, patients must attend regular meetings with a dietician/nutritionist and a psychologist, as well as lose a pre-determined amount of weight to help improve the chances they will be successful with the surgery [20–22]. It is only after the evaluation process that patients can receive an appointment with the bariatric surgeon. Further regulations require that the patient will only meet with the bariatric surgeon if they can be guaranteed to receive their surgery within a year. If the wait extends past 1 year, then the patient must be re-evaluated as co-morbidities may have worsened in this time [18]. As resources for bariatric surgery in Canada are limited, these bottlenecks in the pre-operative process further strain resources and limit access to care [18].

It should be noted that limited private options for bariatric care are available in Canada. The adjustable gastric band, for example, is readily available in private clinics across Canada. These private domestic options often offer shorter wait times than typically experienced in the public system, but at an average cost of $16,000, typically require out-of-pocket payments that are higher than what is required in some medical tourism destinations [23]. Although we know that Canadians are indeed going abroad for bariatric surgery, based on media coverage and internet discussion (e.g., CBC, Global News, National Post) [24–26], there has been no attempt to understand these patients’ first-hand experiences of engaging in this form of medical tourism.

Here we address this knowledge gap by drawing on the findings of 20 qualitative interviews conducted with former Canadian bariatric tourists. Our main objectives are two-fold. First we specifically seek to explore three key challenges experienced by this group that contributed to their pursuit of surgery abroad. These challenges work together to address our research question seeking to better understand the motivating factors that are driving the practice of bariatric tourism by Canadians. Second, based on the analytic findings, we seek to build an understanding of how bariatric tourism by Canadians can be conceptualized as a form of circumvention tourism. As previously outlined, circumvention tourism *typically* refers to procedures that are illegal in the patient’s home country but legal in the destination country. As bariatric surgery is a legal practice in Canada, we help to broaden the understanding of the ways in which the framework of circumvention tourism can be applied to procedures that are indeed legal, but potentially inaccessible to patients due to structural, administrative or regulatory barriers, and therefore driving them to seek the care elsewhere.

## Methods

Using case study methodology, the analysis presented in this paper contributes to a qualitative exploratory study examining Canadian patients’ first-hand experiences with medical tourism for bariatric procedures. In order to fully understand the health care experience and decision making of these Canadian bariatric tourists, a case study is appropriate in order to conduct the intensive study of one particular phenomenon experienced by one particular, usually homogenous, group of people in which context is significant to the phenomenon [27]. Furthermore, as this study was an exploratory study of patient experience, a case study of patient experience is best suited to providing initial insights into this phenomenon [19].

### Recruitment

After obtaining ethical approval from Simon Fraser University’s Office of Research Ethics, we sought to recruit Canadians that had previously travelled outside of Canada to receive bariatric surgery to participate in interviews. As there is no system in Canada that tracks individuals leaving the country each year for medical procedures, social media was used as means to advertise to prospective participants. Targeted advertisements asking prospective participants to contact the principal investigator were placed in popular bariatric surgery support groups on the social media website Facebook as well as other online patient forums. Additionally, similar advertisements were placed on the popular advertisement website Craigslist. A post was also written about the research on a popular Canadian obesity/bariatric surgery blog^2^ and the primary investigator’s contact information was made available for prospective participants. Finally, snowball sampling was used whereby existing participants were asked to share information about the study with others in their networks who were also eligible. This study sought to recruit between 15 and 20 participants. This number was selected based on previous research, in line with case study methodology, demonstrating that saturation, in which no new concepts and ideas emerge, is often achieved between 12 and 20 interviews [28, 29].

Participation in the study required that the participant meet the inclusion criteria. Inclusion criteria mandated that they must: (1) be a Canadian citizen or permanent resident and thereby be eligible for coverage under Canada’s public health plan; (2) have received care privately abroad that was paid for out-of-pocket with no reimbursement from the Canadian health care system; (3) be over the age of 18 at the time of the interview; and (4) have travelled for their respective surgery within the last 10 years. The 10 year window for surgery was chosen in order to capture more recent surgical techniques. As most bariatric procedures are now preformed laparoscopically, older surgical techniques would likely lead to a different experience that would be more presently experienced.

### Data collection

Semi-structured interviews with former Canadian bariatric tourists were conducted by phone from February to May, 2016. These interviews were conducted using a scripted semi-structured interview guide. We elected to use a semi-structured approach in this exploratory study as it allows participants to provide more detail as they see pertinent beyond simply answering the questions directly being asked. The interview guide was developed after a thorough literature review was conducted of the existing medical tourism research, as well as the literature pertaining to bariatric tourism and, more specially, bariatric tourism and the Canadian context. The interviews covered a wide array of topics including: the patient’s reasons for seeking bariatric surgery, their experiences of trying to obtain care domestically, information seeking processes, experiences while obtaining the care abroad, aftercare experiences, as well as reactions/support from family, friends, and members of the Canadian medical system (e.g. family physicians and aftercare specialists, such as dieticians/nutritionists and bariatric surgeons). All interviews were conducted by the same investigator, the lead author of this paper. Each interview ran for approximately 30–50 min.

### Analysis

All interviews were digitally recorded and transcribed verbatim by the primary investigator. Using Braun and Clarke’s articulation, thematic analysis was undertaken to identify, analyze and subsequently, report key themes in the data. A theme is a pattern in the data, identified by many participants, that is relevant to the overall research questions and study objectives [30]. As this is an exploratory analysis of patient experiences with international bariatric tourism, the advantage of Braun and Clarke’s [30] description of thematic analysis is that the analysis not reliant on any pre-existing qualitative theories. Therefore, when used in conjunction with case study methodology and semi-structured interviews, the key themes identified by participants, pertinent to our research objectives, can be identified, analyzed and described. Following Braun and Clarke’s method of thematic analysis, an inductive approach was taken in which the coding of the data was derived directly from the data itself, as opposed to coding within a pre-existing coding frame [30]. Our process of thematic analysis followed six key steps, as per Braun and Clarke (2006). The first step was for each of the authors to familiarize themselves with the data through independent review of the transcripts and to note key emerging concepts and issues brought forth by the participants. The transcripts were then reviewed collectively amongst the team and the independent reviews were compared for overlap [30]. In the next step, the areas of overlap allowed for a codebook of initial codes to be generated. The lead author then reread all the transcripts and coded each in relation to the codebook, adding to and refining the codes as needed. In the third step, patterns in the codes were identified and then grouped into overarching themes. The fourth and fifth steps in our analytic process encompassed reviewing and then refining these overarching themes to ensure no overlap between the themes [30]. From this process, three meta-themes emerged that will be the focus of this present analysis. Finally, in the last step, the lead author then identified excerpts relevant to each theme. The most illuminating or illustrative of each theme was selected by the lead author for use in this analysis which was later confirmed by the other authors. Through involving all the authors throughout the analysis, researcher reflexivity was facilitated through systematically constructing the codes and later the themes together across multiple readings of the transcripts, consequently minimizing the chance of bias being imparted as a result of only one researcher conducting the analysis. In all steps of the analysis, consensus was reached among all the researchers before proceeding to the next step.

## Results

In total, 20 former Canadian bariatric tourists from five of Canada’s 10 provinces were interviewed. All participants were Canadian citizens who qualified for provincially funded health care but not all met the criteria for publicly funded bariatric surgery in terms of the BMI requirements. The type of bariatric surgery pursued abroad also varied amongst participants, although the majority underwent the Vertical Sleeve Gastrectomy (VSG) procedure (*n* = 16). See Table 2 for an overview of key participant demographic characteristics.

**Table:** Table 2

Characteristic	Count
Province of Residence	British Columbia: *n* = 2
	Alberta: *n* = 4
	Saskatchewan: *n* = 12
	Manitoba: *n* = 1
	Nova Scotia: *n* = 1
Age (at time of seeking surgery)	30–40: *n* = 4
	40–50: *n* = 8
	50–60: *n* = 6
	60 or older: *n* = 2
Sex	18 females; 2 males
Destination country for procedure	All participants travelled to Mexico
Type of Bariatric procedure received	Adjustable Lap Band: *n* = 1
	Vertical Sleeve Gastrectomy: *n* = 16
	Gastric Plication: *n* = 2
	Roux-en-Y Gastric Bypass (RNY): *n* = 1

Participants’ motivations to seek care abroad were extensively probed. They repeatedly acknowledged experiencing barriers to accessing care domestically as their main motivations for travelling abroad. Our analysis indicates three types of access barriers: (1) not meeting the body mass index (BMI) requirements to qualify for publicly funded surgery in Canada; (2) structural barriers around which procedures are or are not available locally; and (3) the lengthy pre-operative program required of all surgical candidates in Canada. These three barriers degraded patients’ ability to access bariatric surgery domestically and consequently pushed them to seek bariatric surgery internationally. In this way, bariatric tourism by Canadians can be seen as these patients circumventing the system in Canada. These barriers will be examined in more detail throughout this section. Though we discuss each separately, we acknowledge that many participants experienced more than one domestic access barrier. As we seek to understand first-hand experiences, direct verbatim quotation are provided in this section as a means to let the participants ‘speak’ where possible to the barriers they experienced.

### Structural barriers

Some participants experienced structural barriers to bariatric surgery in their provincial health care systems that they felt were best circumvented by accessing care abroad. By structural barrier we are referring to barriers in the health system that prevented access, such as lacking provincial health care coverage for a *specific* bariatric procedure or the limited availability of experienced surgeons to perform a particular surgery. Every participant indicated having experienced a structural barrier to access that resulted in them seeking care abroad.

At the time of seeking surgery many found that the VSG was not offered locally, or even in their home provinces, despite the fact that most provinces and territories have approved this procedure in their public health care systems, as shown in Table 1 above. For example one participant discussed how she travelled to Mexico for the surgery so that she could have:

*a different version of the surgery than they were offering in Canada, or in my local area, at that time. They weren’t offering the sleeve, which is the VSG, which I got. They were just doing the straight R-en-Y which is a slightly more invasive surgery than the one that I received (Participant #3).*

Another participant stated: “*and then my understanding was that there’s only one surgery that they do [in the province] and it’s the gastric bypass and I wasn’t interested in that one at all” (Participant #12).* Lack of local availability of procedures that participants felt best suited their needs prompted many to seek their desired care elsewhere by circumventing this domestic barrier to access.

The interviews revealed that in some cases participants opted for care abroad due to concerns about the quality of surgery available locally. One participant, for example, expressed concerned over the way the surgery was performed at home:

*initially when I was looking, what I was understanding is that the one surgeon who was doing it was only just starting this laparoscopically and still seemed to have a preference for doing the gastrectomy versus the sleeve, of the full gastroplasty rather (Participant #4)*

Another expressed concern over the types and variety of procedures being offered in Canada.

*I don’t mean to be rude about Canada, but I don’t think it would even be an option [to have surgery at home] because they are so far behind here. They’d be doing something that they did twenty years ago. So that was another part of the reasons that attracted me to Tijuana is these physicians, this is all they do (Participant #13)*

She felt constrained in regard to her surgical choices and did not feel she could get quality surgery with the current surgical techniques she desired anywhere in Canada, thus prompting her to seek care abroad.

### Body mass index (BMI) requirements

As discussed earlier, Canada follows the recommendations from the National Institutes of Health (1991) that indicate that in order to qualify for surgery a patient must have a BMI of 40 kg/m^2^ or higher or a BMI of 35–39.5 kg/m^2^ with the presence of one or more significant co-morbidities, such as type II diabetes, hypertension, and/or obstructive sleep apnea. Many participants cited these BMI cut-off points as a barrier to accessing care domestically, referring to them as very strict requirements with little room for consideration of other factors, such as lifetime history of obesity. Thirteen of the twenty participants interviewed explicitly discussed their lower-than-required-BMIs as the primary barrier to accessing bariatric surgery in Canada. One participant discussed how close her BMI was to the requirement but that it was not close enough to qualify her for surgery: “*At the time I was 34 [BMI], but I didn’t qualify, I wasn’t fat enough or my BMI wasn’t high enough to even be looked at for bariatric surgery in Saskatchewan.”(Participant #6).* In this case, while the participant’s BMI was quite high she did not have other significant co-morbidities to qualify her even for the lower BMI requirement.

For participants who tried to work with their primary health care physicians to get into the queue for surgery, BMI requirements limited the extent to which physicians could assist with facilitating approval for surgery. For example, one participant expressed frustration with her primary health care provider’s lack of ability to get her into the queue:

*They [doctor] said your BMI’s not high enough. Your BMI has to be at least 35 and I think I was 32 or something. So they said nope, you’re not high enough. And then my weight kept going up and I said ‘oh great, this is nice and now I have to see my weight keep going up’. But it wasn’t 35, it was 33 or 34 so it was close to 35 and they couldn’t refer me and I couldn’t do anything because my BMI wasn’t high enough (Participant #17).*

Another participant, felt generally frustrated over not being able to access care because of her weight: “*okay you’re not going to help me because I’m not fat enough?! That doesn’t make any sense.” (Participant #6).* Participants also expressed frustration with the medical advice they received when their BMIs were not high enough. For example, one participant expressed concern over a conversation she had with their family doctor: “*He had said that because I was only obese, like I wasn’t morbidly obese, that it [surgery] wouldn’t really be an option”(Participant #16).* In this case, she was instructed that in order to qualify for surgery she “*would either need to put on a bunch of weight or take off a bunch of weight on my own. There was no in-between, nothing they could really do to help me just because I was just obese.” (Participant #16).* With a history or failed attempts at conventional methods of diet and exercise, this participant felt Canada left her with no domestic options to address her weight that would work for her.

Not all participants chose to approach their primary care physicians about their desires to access bariatric surgery in Canada. Either through their own research or through word-of-mouth/anecdotal stories, some knew they would not qualify for the BMI requirements and therefore did not even attempt to seek the care in Canada. For example, one participant explained how “*I knew I’d never be a candidate in Canada ‘cause I didn’t have enough weight to lose.” (Participant #13).* While another stated: “*I don’t think I could have got it done in Canada. I don’t think I was big enough for their, for that.” (Participant #10).* Both of these participants dismissed Canada’s public health care system as even being an option for them to seek bariatric surgery based on the knowledge of their own BMIs and consequently went straight to researching care abroad.

### The pre-operative process in Canada

Finally, the third barrier to accessing bariatric surgery in Canada as reported by the participants was the lengthy pre-operative process that added significantly to the surgical wait time. Twelve of the twenty participants interviewed explicitly discussed the pre-operative process and the consequent wait time as a barrier to seeking the care in Canada. For many, knowledge of the wait time for surgery in Canada posed significant concern. All participants acknowledged the negative state of health they were in prior to surgery and expressed a deep desire to take action to reduce weight-related health issues despite failed attempts to do so through conventional diet and exercise. Consequently, the wait time for surgery was seen as a time-draining domestic requirement they opted to circumvent by seeking care abroad. For example, one participant stated:

*Well, I didn’t even look into anything in Canada because for one thing I could get it done right away [in Mexico]. Whereas I know in Canada it takes up to five years. Like I had it done within a month and a half after I starting looking for, in, in Mexico (Participant #5).*

For many of the participants, this knowledge of the wait time prevented them from even beginning the process to seek approval for surgery domestically.

Another aspect of the pre-operative process in Canada that some participants found concerning were the multiple steps it required, such as losing a pre-determined amount of weight and consulting with multiple specialists including a dietician/nutritionist, a psychologist and the bariatric surgeon prior to surgery being scheduled. As one participant explained:

*Well the thing is, like I’ve read several things about the process in Canada and you know they give you a diet and see whether you can follow that procedure for a year and then there’s some emotional, some counselling for another couple of years, and then, you know, wait another year and see whether you still want to do it or not. There’s lot of pre-scanning that’s done before you even get a chance to get in* *(Participant #5)* *.*

Another participant echoed similar sentiments:

*So I was going to have a three year wait to get it done and I wasn’t willing to wait that long because you have to go through all the steps. Go through your dietician and then you gotta see a psychiatrist and then you gotta meet with the surgeon and then they’re going to decide if you’re a, if you can do the surgery, right?(Participant #15).*

The significant number of steps that had to be successfully accomplished in order to be approved for surgery in addition to the depth of involvement required by some steps, specifically the pre-surgery weight loss requirement, was seen as an unnecessary barrier to care by most participants.

Some participants expressed concern that a Canadian patient could spend years trying to prove they are a good candidate for bariatric surgery, only to be denied, and that they were not willing to expose themselves to this possibility. Circumventing this process became attractive, if not necessary. As one participant stated:

*I didn’t have to prove to anybody that I was a good candidate [to privately have surgery in Mexico] other than health-wise, that I could handle doing the trip and that I could handle loosing 70 or 80 pounds... So I kind of made that decision on my own that I was going to do this. Whereas, going through the procedure that’s in Canada, there’s a rift there of someone saying you’re not psychologically ready for it (Participant #5).*

By circumventing this process, participants felt they were more empowered in their own health care decision-making. They did not have to navigate the possibility of being rejected for the surgery after years of waiting in the queue, as well as lost time and energy spent in multiple appointments with specialists. Pursuing the care abroad consequently was viewed as necessary to ensuring that desired bariatric care was indeed obtained.

## Discussion

In this analysis we have identified three main barriers to access Canadians have faced when attempting to access bariatric surgery domestically. These barriers included: 1) strict BMI requirements to qualify for publicly funded surgery; 2) structural barriers limiting local access to desired surgeries; and 3) the pre-operative process that could stretch across multiple years and required steps. Collectively and individually these barriers serve as push factors that encourage some Canadians to seek bariatric surgery abroad, which was demonstrated in the previous section. The ability of Canadians to access bariatric surgery abroad creates potential opportunities for accessing weight-related health care that some patients identify as necessary and urgent while the Canadian health care system may not. As shown throughout our findings section above, by engaging in this form of medical tourism this behaviour can be viewed as Canadians circumventing the regulations, processes, precautions, requirements, and restrictions built into their domestic health care system.

As we noted at the outset of the findings section, most participants experienced multiple barriers to accessing bariatric surgery domestically in Canada, with some experiencing all three of those identified here. For example, the restrictions for care in Canada, including both not meeting the BMI criteria and the lengthy pre-operative process, led several participants to go abroad for care. It was also common for participants to not meet the BMI criteria for domestic surgery while simultaneously questioning the quality of surgical options in Canada and the length of the pre-operative wait time. In such cases these barriers – both perceived and realized – acted together in prompting consideration of international surgical options. The presence of such barriers and the ways in which multiple barriers interacted in order to shape one’s access to surgery domestically facilitated, if not drove, participants to circumvent domestic options in favour of privately purchasing bariatric surgery abroad. Furthermore, in Canada primary care physicians act as gatekeepers to more specialized care [31]. For many of the participants, not being able to get a referral from a primary health care physician to a surgeon left them with few options for having the surgery in Canada and consequently these individuals felt they had no choice but to circumvent the system and seek care abroad.

By examining participants’ experiences with attempting to gain access to bariatric care in Canada, it is apparent that many felt the system was not helping them to address their weight-related health concerns. Here we see a parallel to patients who engage in unproven treatments such as stem cell therapies or chronic cerebrospinal venous insufficiency (CCSVI) treatment via medical tourism. In the case of CCSVI treatment and other stem cell therapies, studies have shown that patients are engaging in these treatments abroad as a result of a loss of hope for and trust in the care that can be received domestically [32]. Similar sentiments were echoed by the participants in this study, especially in regard to what many characterized as a hopeless pre-evaluation process in which both the strict BMI requirements and repeated evaluations had to be met in order to qualify for bariatric surgery domestically. In the meantime, throughout this period obesity-related co-morbidities would likely be worsening and, in some cases, they could get to the point of negatively affecting surgical outcomes [15, 33, 34]. The length of time this process could persist, coupled with low surgical capacity for bariatric procedures across Canada [16], left many participants with similar feelings of a loss of hope for and trust in the Canadian health care system. In the context of this type of circumvention tourism, participants sought hope through treatment abroad. While doing so addressed the immediate perceived need for medical intervention, exiting the Canadian system did nothing to address the loss of trust in the domestic health care system. This is concerning given that patients’ health outcomes and continuity of care are best when they form a trusting relationship with their health care providers and also have trust in the quality of care made available [35, 36].

As discussed in the background section, traditionally the label of circumvention tourism has been applied to procedures that are illegal in the patients’ home country, such as assisted suicide, abortion, commercial surrogacy, and unproven stem cell interventions [7]. Due to their illegal nature and the fact that they often raise moral concerns, these practices have sometimes been described as presenting the ‘darker’ side of medical tourism [37]. However, as this paper has shown, procedures with strict regulatory restrictions can also lead to similar circumventing behaviour. In the same way, moral concerns may also be raised both by the presence of the structural barriers discussed in this analysis and in the participants actions to circumvent them. However, deeper critical examination of the moral concerns associated with these barriers is beyond the scope of this analysis.

In this way circumvention tourism can also be conceptualized as including procedures that are legal but unavailable in the medical tourist’s home country, such as bariatric procedures in Canada. This analysis has shed light on the specific access barriers Canadians are looking to circumvent in seeking bariatric surgery abroad – and specifically in Mexico. A concern, however, is that by circumventing such regulations and restrictions, participants were also leaving behind the processes put in place to protect their health and safety. For example, there is an extensive literature that demonstrates the utility of pre-operative weight loss and counselling in supporting long-term surgical weight loss success [38, 39], and it is clear that Canada’s guidelines have a basis in such research. As with other forms of circumvention tourism, in the context of Canadians’ pursuit of bariatric surgery abroad, such patients may not be aware of why certain protections have been put in place or the implications of their avoidance.

Restrictions and regulations regarding access to bariatric surgery help to control costs and resource allocation in the context of the finite resources available in Canada’s public health care system [40, 41]. Though patients exiting the Canadian system via circumvention tourism are paying for such care privately, research has shown that this practice places further strain on the Canadian health care system once bariatric tourists return home and require aftercare or care for potential complications [40, 41]. For example, in one bariatric weight management clinic alone, 62 medical tourists imposed additional health care spending by the province of Alberta of CDN$1,834,168. The majority of these costs were be attributed to those needing extensive corrective surgery and requiring hospital stay due to complications arising following return from surgery abroad [40]. It was also found that when medical tourists presented to the clinic with complications they were potentially taking already very limited resources from those waiting to receive the surgery domestically [41]. Therefore, bariatric tourists engaging in circumvention tourism can be seen as further entrenching and perpetuating the barriers restricting them from obtaining access to care domestically in the first place. This was not, however, a position taken by any of the participants in this study. It is also important to note that, while not a specific focus of this analysis, the participants in this analysis experienced a very low rate of serious complications (*n* = 2) requiring hospitalization. This low rate of serious complication, however, may not reflect a typical complication rate amongst bariatric tourists and may be a characteristic of participants’ willingness to participate in the study.

Through an examination of Canadian patients’ experiences of international bariatric tourism, we have met our second objective through showing how broadening conceptualizations of circumvention tourism can allow examination of different cases of medical tourism under a framework of circumvention. In the context of the current analysis, we showed how participants were circumventing regulations and restrictions rather than legal barriers. While in this case many of the traditional ethical implications that have been conceptualized in the mainstream circumvention tourism literature that we summarized in the introduction did not apply, the actions of circumventing the domestic barriers by going abroad for privately-funded medical care were mirrored. In light of the findings of this study, future research should examine the implications the practice of circumvention tourism by Canadian bariatric tourists has on the Canadian health care system beyond financial consequences. These could include: 1) bariatric patient trust in the Canadian health care system in situations of bariatric tourism; 2) changes in provincial surgical capacity and any corresponding changes in bariatric tourism by Canadians;3) the use of Canadian health care resources both pre-and post-operatively when bariatric tourists are circumventing the pre-operative process required in Canada; and 4) if the cost associated with private bariatric care in Canada also act as a prohibitive factor to domestic access and if it is also driving circumventive behaviours. Several participants discussed looking into private options in Canada, but as the cost of private care options in Canada was beyond the scope of this analysis, it is not discussed here. The three regulatory and administrative barriers identified in this study included the BMI requirements, structural barriers in regard to procedure availability, and the lengthy pre-operative process required in Canada. Throughout the study, the authors collaborated on design, data collection and analysis, therefore enhancing reflexivity and data triangulation on the part of the researchers. Through this process reasonable transferability of research findings was achieved to aid future research. Future examination of these barriers, both in regard to their implications *on* the practice bariatric tourism by Canadians and the implications *of* the practice of bariatric tourism on Canadian health care will enhance understanding of this relatively unstudied area of health mobility.

### Limitations

Difficulty in recruitment for the study, as we sought to interview a highly stigmatized population, led to snowball sampling being heavily relied on. Consequently it is possible that individuals had similar experiences due to shared or similar characteristics and overlapping social networks. For example, as the majority of participants were living in Canada’s western provinces, they may have experienced similar barriers in access to care due to geographic location. In the same way, this analysis may not have captured more negative experiences due to a lack of willingness to participate among those who do not have a positive experience or a greater level of stigmatization. Additionally, all participants received their respective bariatric surgeries in Mexico and so we captured no diversity in terms of destination location and experience. It is also important to note that no participants lived in Canada’s most populous province, namely Ontario. Barriers to domestic bariatric surgery may be quite different for residents of Ontario as the province has increased its surgical capacity in recent years [15, 16].

## Conclusion

Here we have presented the findings of interviews with 20 former Canadian bariatric tourists, specifically examining the domestic barriers to care experienced by those who opted to go to Mexico for bariatric surgery. Three main challenges emerged that appear to be serving as push factors motivating Canadians to seek bariatric surgery abroad: (1) not meeting the BMI requirements for surgery through public funds in Canada, (2) structural barriers limiting access to specific procedures and/or in particular locations, and (3) lengthy and multi-stepped pre-operative processes that can leave people stuck at various points in the queue while waiting for care. In many cases participants experienced multiple barriers, only heightening the frustration they experienced while trying to access care domestically while also lessening their trust in the Canadian health care system.

Traditionally circumvention tourism has been conceptualized to encompass procedures sought abroad that are illegal in the medical tourist’s home country, and sometimes even in the destination country. However, by examining bariatric tourism through a circumvention framework, we have shown how regulations that restrict access to fully legal care at home can push people abroad in order to obtain desired care that is inaccessible domestically. We now have a better understanding of the practical limitations of current conceptualizations of circumvention tourism. Through expanding this framework to include cases of patients circumventing structural, administrative or regulatory barriers built into the system we can ask questions beyond those that are more legal or moral in nature. More specifically, the fundamental concerns we can now potentially address could pertain to issues around continuity of care, disruptions in care, and potentially the usefulness of the barriers in the first place. We encourage future research in the medical tourism field to examine the role of inaccessibility in driving private pursuit of care abroad as well as the ways in which inaccessibility and illegality co-exist in shaping how people select destination countries through the practice of circumvention tourism.

## Abbreviations

BMI: Body Mass Index
RNY: Roux-en-Y Gastric Bypass
VSG: Vertical Sleeve Gastrectomy
WHO: World Health Organization

## Footnotes

1 The 1% surgical output versus eligibility statistic reflects the fact that not all individuals that are classified as obese will opt to seek surgery as a solution to their weight-related health, in addition to limited surgical capacity for bariatric surgery across Canada and the multiyear evaluation process required to access care.

2 The blog is Dr. Sharma’s Obesity Notes out of Alberta, Canada.

## References

[1] Connell J (2013). Contemporary medical tourism: conceptualisation, culture and commodification. Tour Manag.

[2] Snyder J, Crooks VA, Johnston R (2012). Perceptions of the ethics of medical tourism: comparing patient and academic perspectives. Public Health Ethics.

[3] Fisher C, Sood K (2014). What is driving the growth of medical tourism. Health Mark Q.

[4] Lunt N, Mannion R (2014). Patient mobility in the global marketplace: a multidisciplinary perspective. International Journal of Health Policy and Management.

[5] Sethna C, Doull M (2012). Accidental tourists: Canadian women, abortion tourism and travel. Women’s Studies.

[6] Crooks VA, Kingsbury P, Snyder J, Johnston R (2010). What is known about the patient’s experience of medical tourism? A scoping review. BMC Health Serv Res.

[7] Cohen IG (2015). Patient with passports: medical tourism, law and ethics.

[8] Charo RA (2016). On the road (to a cure?) — stem-cell tourism and lessons for gene editing. N Engl J Med.

[9] McGuinness S, McHale JV. Transnational crimes related to health: how should the law respond to the illicit organ tourism? Leg Stud. 2013, 34(4):682–708.

[10] Bergmann S (2011). Fertility tourism: circumventive routes that enable access to reproductive technologies and substances. Signs.

[11] Brown MG (1993). Rationing health care in Canada. Annals of Health Law.

[12] Owen-Smith A, Donovan J, Coast J (2015). How clinical rationing works in practice: a case study of morbid obesity surgery. Soc Sci Med.

[13] Statistics Canada: Overweight and obese adults (self-reported). 2014. http://www.statcan.gc.ca/pub/82-625-x/2015001/article/14185-eng.htm. Accessed 5 Oct 2015.

[14] Gastrointestinal Surgery for Severe Obesity: NIH Consens Statement Online 1991, 9(1):1–20. 1760489

[15] Christou NV, Efthimiou E (2009). Bariatric surgery waiting times in Canada. Can J Surg.

[16] Canadian Institute for Health Information: Bariatric Surgery in Canada. https://www.cihi.ca/en/growingdemand-for-bariatric-surgery-challenges-patients-system. Accessed 20 Oct 2015.

[17] Kim DH, Sheppard CE, de Gara CJ, Karmali S, Birch D (2015). Financial costs and patients' perceptions of medical tourism in bariatric surgery. Can J Surg.

[18] Christou NV (2011). Access to bariatric (metabolic) surgery in Canada. Can J Diabetes.

[19] Suter WN (2012). Qualitative data, analysis, and design. Introduction to educational research: a critical thinking approach.

[20] Buchwald H, Ikramuddin S, Dorman RB, Schone JL, Dixon JB (2011). Management of the metabolic/bariatric surgery patient. Am J Med.

[21] Halloran K, Padwal RS, Johnson-Stoklossa C, Sharma AM, Birch DW (2011). Income status and approval for bariatric surgery in a publicly funded regional obesity program. Obes Surg.

[22] Runkel N, Colombo-Benkmann M, Hüttl TP, Tigges H, Mann O, Sauerland S (2011). Bariatric Surgery. Dtsch Arztebl Int.

[23] Martin AR, Klemensberg J, Klein LV, Urbach D, Bell CM (2011). Comparison of public and private bariatric surgery services in Canada. Can J Surg.

[24] Pagel J, Berry S: Bariatric medical tourism comes at a high cost for patients and Canada. CBC radio- the Current; 2016. http://www.cbc.ca/radio/thecurrent/the-current-for-march-17-2016-1.3495265/bariatric-medicaltourism-comes-at-a-high-cost-for-patients-and-canada-1.3495273. Accessed 21 Mar 2016.

[25] Goh S: Weight-loss tourism costing Alberta taxpayers thousands to fix medical mistakes. Global News Edmonton; 2016. http://globalnews.ca/news/2597125/weight-loss-tourism-costing-alberta-taxpayers-thousandsto-fix-medical-mistakes/. Accessed 23 Mar 2016.

[26] Kirkey S. Fixing botched stomach-shrinking surgeries performed in other countries costs Canadians millions. In: The National Post; 2016. http://news.nationalpost.com/health/0310-na-bariatric. Accessed 9 Mar 2016.

[27] Baxter P, Jack S (2008). Qualitative case study methodology: study design and implementation for novice researchers. Qual Rep.

[28] Guest G, Bunce A, Johnson L (2006). How many interviews are enough? An experiment with data saturation and variability. Field Methods.

[29] Mason M. Sample size and saturation in PhD studies using interviews. Forum: Qualitative Social Research. 2010;11(3)

[30] Braun V, Clarke V (2006). Using thematic analysis in psychology. Qual Res Psychol.

[31] Chan BTB, Austin PC (2003). Patient, physician, and community factors affecting referrals to specialists in Ontario, Canada: a population-based, multi-level modelling approach. Med Care.

[32] Snyder J, Adams K, Crooks VA, Whitehurst D, Valle J (2014). **“**I knew what was going to happen if I did nothing and so I was going to do something”: faith, hope, and trust in the decisions of Canadians with multiple sclerosis to seek unproven interventions abroad. BMC Health Serv Res.

[33] Lakoff J, Ellsmere J, Ransom T (2015). Cause of death in patients awaiting bariatric surgery. Can J Surg.

[34] Gregory DM, Newhook JT, Twells LK (2013). Patients’ perceptions of waiting for bariatric surgery: a qualitative study. Int J Equity Health.

[35] Birkhauer J, Gaab J, Kossowsky J, Hassler S, Krummenacher P, Werner C, Gerger H (2017). Trust in the health care professional and health outcome: a meta-analysis. PLoS One.

[36] Lee Y, Lin JL (2008). Linking patients’ trust in physicians to health outcomes. Br J Hosp Med.

[37] Cooper M, Hieda M, Cooper M, Vafadari K, Hieda M (2015). The darker side of medical tourism? End of Life Choice, Human Trafficking, and Organ Transplants. Current Issues and Emerging Trends in Medical Tourism Hershey: Medical Information Science Reference.

[38] Neff KJ, Olbers T, le Roux CW (2013). Bariatric surgery: the challenges with candidate selection, individualizing treatment and clinical outcomes. BMC Med.

[39] Breznikar B, Dinevski D (2009). Bariatric Surgery for Morbid Obesity: Pre-Operative. Assessment, Surgical Techniques and Post-Operative Monitoring. J Int Med Res.

[40] Sheppard CE, Lester EL, Karmali S, de Gara CJ, Birch DW (2014). The cost of bariatric medical tourism on the Canadian healthcare system. Am J Surg.

[41] Sheppard CE, Lester EL, Chuck AW, Kim DH, Karmali S, de Gara CJ, Birch DW (2014). Medical tourism and bariatric surgery: who pays?. Surgical Endoscopy and Other Interventional Techniques.

